# Multimodal Assessment in Clinical Simulations: A Guide for Moving Towards Precision Education

**DOI:** 10.1007/s40670-024-02221-7

**Published:** 2024-11-19

**Authors:** Deborah Schwengel, Ignacio Villagrán, Geoffrey Miller, Constanza Miranda, Serkan Toy

**Affiliations:** 1 Anesthesiology and Critical Care Medicine, Johns Hopkins University School of Medicine, Baltimore, MD USA; 2 Health Sciences Department, Faculty of Medicine, Pontificia Universidad Católica de Chile, Santiago, Chile; 3 Computer Science Department, School of Engineering, Pontificia Universidad Católica de Chile, Santiago, Chile; 4 Department of Emergency Medicine, Johns Hopkins School of Medicine, Baltimore, MD USA; 5 Johns Hopkins School of Nursing, Baltimore, MD USA; 6 Johns Hopkins Medicine Simulation Center, Johns Hopkins School of Medicine, Baltimore, MD USA; 7 Biomedical Engineering Department, Johns Hopkins University, Baltimore, MD USA; 8 Department of Basic Science Education, Virginia Tech Carilion School of Medicine, Roanoke, VA USA; 9 Department of Health Systems and Implementation Science, Virginia Tech Carilion School of Medicine, Roanoke, VA USA

**Keywords:** Multimodal assessment, Simulation-based learning, Precision education, Multimodal learning analytics

## Abstract

Technological advances and cutting-edge data analytics approaches give new prospects for using multimodal assessments in simulation-based medical education. These multimodal approaches consider the interaction of data obtained from multiple sources and can enhance our ability to assess the educational efficacy of simulations and optimize learning experiences for each individual learner. However, several challenges could jeopardize the successful implementation of this approach. We present a practical guide for integrating multimodal assessment in clinical simulation-based education, offering educators and researchers a systematic checklist to consider before undertaking these multimodal assessment initiatives.

## Introduction

Health sciences schools have widely adopted clinical simulations as an effective method for teaching clinical competencies [1–3]. Observational measures such as global scales or checklists are commonly used to assess simulated clinical performance [4, 5]. However, new assessment paradigms are now conceivable thanks to the recent progress in simulation technology, wearable sensors, and machine learning (ML) and artificial intelligence (AI) approaches [6].

Novel assessments can incorporate objective data sources for a comprehensive examination of factors influencing learner performance using simulator-generated metrics (including manikin-based as well as virtual and mixed-reality simulation models), video and motion capture technologies, and wearable sensors [7–10]. These technologies and ML approaches allow non-invasive data acquisition and analysis of neurophysiological and biomechanical markers, e.g., electroencephalography [11, 12], motion capture data [13–15], and AI-based image or video analysis [16].

It is still increasingly important to incorporate robust observational and self-report measures with good psychometric properties, including variables of cognitive load and other psychosocial factors that may provide insights into learning and clinical performance [17–19]. The incorporation and triangulation of multiple data sources will likely increase the accuracy of the assessment and optimization of the learning process for each individual learner, ideally leading to “precision education” [20]. Just as precision medicine should improve patient care outcomes, precision education should optimize the learning process and outcomes for the individual through the continuum of medical education, using data science and technology [21–23].

Fast-evolving technology-oriented approaches to education provide unprecedented opportunities for detailed examination of the learning process while simultaneously presenting new challenges. We must develop efficient methods to obtain integrated data streams to improve learning processes [24]. To this end, learning analytics (LA) has become a popular concept. LA is a multidisciplinary effort that centers on measuring, analyzing, and synthesizing data about learners and the learning environment to better understand and optimize the learning process [25]. When data is obtained from different modalities, multimodal learning analytics (MMLA) emerges. MMLA expands LA methodologies by considering the interaction of data obtained from multiple data channels and sources (i.e., video, logs, audio, gestures, biosensors) [9, 26].

Although still relatively rare, few studies have demonstrated that multimodal assessments enhance evaluation processes in simulation-based training [27–29]. For instance, procedural skills can be objectively assessed using sensors embedded in mannequins, automatically detecting tasks-specific metrics such as timeliness and accuracy of placing oxygen masks, administering medication, and performing CPR. In addition, learner-specific data, including spatial positioning in clinical settings and physiological information from wearable sensors (e.g., electrodermal activity and acceleration), can be correlated with video and audio recordings of interactions, communication, and self-reported activities [27, 30]. Furthermore, the literature suggests that LA holds significant potential for bridging the gap between computational theories and practical challenges in medical education, particularly in feedback processes [29]. Examples include delivering real-time feedback to learners through signaling mechanisms and developing LA interfaces that provide insights into team performance [28, 29]. These interfaces can present feedback using graphics, text, alerts, or haptic signals, complementing the human element in the feedback process.

Using these analytical multimodal approaches can enhance the effectiveness of simulation-based learning. However, it is imperative to consider various challenges before implementing a simulation scenario with multimodal assessment. We provide practical tips to guide the successful implementation of multimodal assessment efforts in clinical simulation-based education. Although some tips have a logical sequence, we do not intend to present them in a definite order.

## Identify a Challenge Within an Educational Context That Requires a Multimodal Assessment

- Tip 1: Establish a clear conceptual framework from the beginning

A successful educational scholarship project begins with a well-defined, significant educational gap or problem. To obtain the desired outcomes, one must make a concerted effort to gain a clear understanding of the problem. This involves conducting general and targeted needs assessments and establishing specific, measurable learning objectives. This can be done by following the steps of Kern’s approach to curriculum development [31]. Identifying an educational problem and formulating a research question can involve reviewing established evidence in a general population and refining it through a targeted needs assessment [32]. To effectively address gaps in learning outcomes, it is crucial to thoroughly understand the learner group and educational context. A targeted needs assessment can help account for variations in the trainees’ level of clinical experience and their interactions with the learning environment, fellow learners, and faculty. A thorough knowledge of these factors builds the foundation for designing the simulation scenario, selecting the most appropriate assessment methods, allocating resources rationally, and assembling a solid research team [33, 34].

- Tip 2: Conduct a thorough literature review

It is not desirable to invest all the time and effort to address a problem that has already been solved and reported in the literature. Staying current with new developments can help save time and resources, especially now that tools such as artificial intelligence are accelerating the advance of techniques and technology in these contexts. When designing the multimodal assessment, it is essential to select metrics based on the literature, research question, context, and objectives. This approach ensures that the metrics provide valuable information while preventing the accumulation of excessive and noisy data and technology prototypes that do not align with research objectives and research team expertise [26, 28]. This may involve a combination of qualitative and quantitative data collection methods. A theory-based and literature-informed process for selecting variables will facilitate hypotheses-driven research. This will enable focusing on the best metrics for testing those hypotheses and rigorously planning the process of obtaining, processing, and analyzing pertinent data.

## Detail a Consistent Plan Considering Multiple Factors and Approaches

- Tip 3: Assemble a well-functioning multidisciplinary team

Integrating multiple assessment modalities in clinical simulations requires a highly motivated team with diverse and complementary skills [26, 28, 35]. Clinical experts, simulation-based education experts, and instructional designers must be involved early in the research design to help build a sound theoretical framework. Additionally, a human factors expert can provide insights into learner needs and behaviors. It may also be necessary to include experts in various methodological approaches. Quantitative and qualitative approaches, for instance, require substantially different methodological expertise, and for a mixed-methods study, both may be needed. On the other hand, if a study involves neurophysiological variables, expertise in using sensors and devices and applying data science may be needed for data acquisition, wrangling, and integration. Finally, experts in educational psychology and psychometrics should also be consulted on assessment instruments to ensure valid and reliable results [35]. Also, given the interdisciplinary nature of MMLA, its implementation requires integrating different theoretical and practical models, such as psychological, physiological, educational, and computational [26, 36]. It is important that all the members of this multidisciplinary team bring a coherent approach to the research plan and execution, including the data collection and processing, as well as the computational assessment and data analysis. They must all be present from the planning stage to incorporate their expertise and perspectives to avoid obvious missteps.

- Tip 4: Consider alignment between research objectives and assessment framework and ensure ethical practice

As multimodal learning analytics (MMLA) becomes increasingly integrated into health professions education, it is essential to carefully align research objectives with a well-formulated assessment framework while addressing the ethical implications of using such data. When designed with clear research goals, multimodal assessments can contribute to research and continuous curricular improvement.

First, ensure the educational problem, research objectives, and assessment framework are well-formulated and aligned. A storyboard for the critical actions can help facilitate this process, including a timeline for when they will be done and by whom. This includes coordinating schedules for trainees, instructors, and simulation staff and managing equipment availability.

Second, securing Institutional Review Board (IRB) approval for any MMLA studies is crucial, particularly when handling sensitive or protected information such as biometric, neurophysiological, or behavioral data. IRB approval ensures that ethical standards are maintained, including informed consent, data anonymization, and compliance with regulations protecting participant privacy. The literature increasingly emphasizes the need to address privacy and ethical concerns when dealing with such complex data sources [37]. Although educational research using neurophysiological and biomechanical markers is not intended to diagnose or treat medical conditions, researchers should be prepared for unexpected findings. These may include neurophysiological markers that may reveal previously undetected health conditions. Researchers should have procedures in place to handle these ethically while protecting participant confidentiality and providing appropriate resources or referrals as needed. Moreover, researchers should implement robust data management procedures to ensure data integrity. This includes secure storage, clear protocols for anonymizing data, and maintaining the accuracy of the analysis. Thoughtful data management not only safeguards participants but also enhances the reliability of research findings, supporting reproducibility and transparency.

If the multimodal assessment is primarily research-focused, employing solid research methods is essential. This includes using assessment instruments with proven reliability and validity, establishing a clear data collection process, and ensuring a sufficient sample size to answer the research question. Although the focus may be on advancing research, these studies often yield critical insights that can improve educational practices, especially in simulation-based learning environments. For example, identifying common learning errors or performance gaps can inform future simulation designs and feedback strategies.

### Teaching Note

It is important to determine whether the multimodal assessment is being developed for research or educational purposes, but ideally, it should be both. A rigorous multimodal assessment will likely offer valuable insights to inform educational practices. Therefore, all research-related critical actions should be planned with educational critical actions and integrated well to achieve good educational practices and high research quality. If the multimodal assessment is focused on education with the provision of feedback to the learner, thought should be given to how actionable the feedback is and whether it truly enhances the learning and experience of the learners.

- Tip 5: Define outcome measures and data collection methods

To conduct impactful research in simulation-based medical education involving multimodal data, it is crucial to define educational objectives and research questions based on a thorough literature review, learner’s needs, learning environment, and context. Specific, measurable objectives encompassing cognitive, affective, and psychomotor domains enable the investigator to select the most suitable metrics for addressing the research question. When possible, use established and standardized measures and instruments to ensure data consistency and comparability across different populations and studies. After creating the hypotheses and determining metrics, a coherent methodology and sequence of events will need to be established. To achieve this, consider creating a schematic outline that aligns outcome measures to hypotheses and a timeline for administering each measure.

**Fig. 1:**
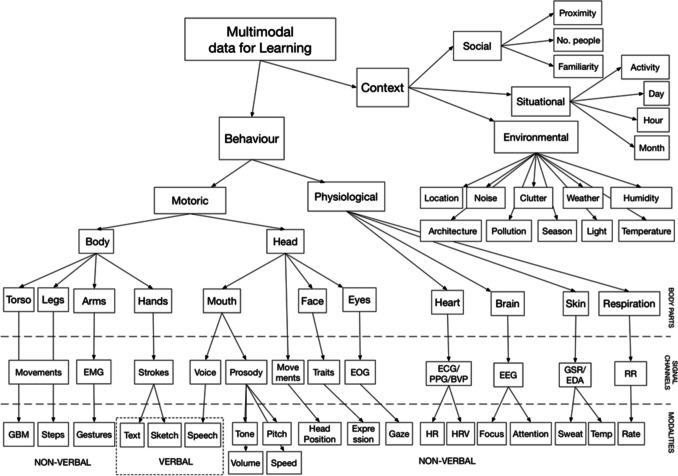
Taxonomy of multimodal data for learning (presented with permission from Di Mitri et al., 2018). EMG, electromyogram; GBM, gross body movement; EOG, electrooculogram; ECG, electrocardiogram; PPG, photoplethysmography; BVP, blood volume pulse; HR, heart rate, HRV, heart rate variability; EEG, electroencephalogram; GSR, galvanic skin response; EDA, electrodermal activity; RR, respiration rate

Exploring the pertinent multimodal data options for addressing the research question is an essential early step in the project design process. Consider multiple data collection modalities, such as video and audio recordings, surveys, and live observation notes, to capture a wide range of information about the educational process and outcomes. Figure 1 illustrates the various data sources that can be used to examine the learning process comprehensively. Learner self-report questionnaires can be used to measure psychosocial variables, such as workload, anxiety, and stress. Observational performance assessment instruments, like checklists, must be carefully selected. It is imperative to provide evidence for the validity and reliability of self-report and observational measures [38]. Neurophysiological data, like attention, engagement, and stress, may include technologies for eye-tracking, electroencephalography (EEG), heart rate variability, etc. Some simulators can provide objective metrics related to performance indicators. It is ideal to use automatized, more efficient techniques, where possible, to eliminate the need to rely only on human raters and reduce bias and subjectivity. As simulation technology and wearable devices rapidly advance, staying current with what is available is essential. What is impossible today may soon become routine practice. New technologies and methods can support research progress and enhance traditional outcome measures. However, using complicated tools and metrics may impose an additional burden on limited resources; thus, simple, feasible methods should be prioritized if they suffice in answering the research question.

### Teaching Note

When designing multimodal educational assessments, select standardized measures to ensure consistency and prioritize simple, feasible methods that effectively capture learner performance. Integrating multiple data sources, such as video, audio, and simulator-generated metrics, can provide a holistic understanding of learner skills and streamline data collection and analysis.

## Define the Educational Resources Required for Implementation

- Tip 6: Design the simulation scenario

**Fig. 2:**
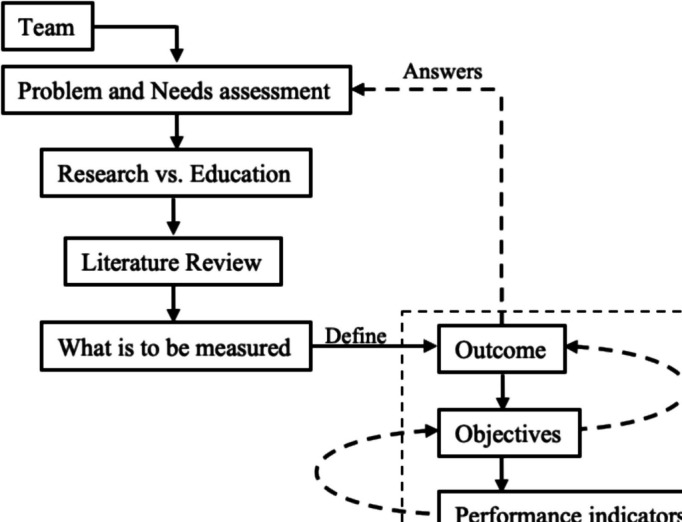
Diagram with initial steps to incorporate multimodal assessments into simulations

The scenario design should allow the trainees and research team to achieve the desired outcome(s). The identified problem, defined objectives, and selected methodology must coalesce into an algorithmic approach (Fig. 2). Upon designing the scenario, it is advisable to review these items to verify coherence and ensure that the objectives are feasible. The scenario should incorporate opportunities to allow streamlined data collection (e.g., EEG signals, self-reported questionnaires, performance) without disrupting the realism and flow of simulation scenarios. Pilot testing the scenarios and study protocol can help identify what is working and what is not so that adjustments can be made to ensure optimal research outcomes. Feasibility and pilot testing can highlight the unexpected failures of data capture, scenario production problems in a specific learning environment, and issues related to learner groups. When using new technologies, time and consideration should be given to device calibration, data synchronization, and hardware and software integration [39]. Selecting the best time to administer questionnaires is also essential to avoid overburdening learners or keying them into the topic of the simulation if it is meant to be an unknown. Determine the advantages of collecting surveys onsite (completion rates) vs. disadvantages (adding to cognitive load, alerting trainees to the topic of study).

- Tip 7: Establish a standard-setting process

A sound standard-setting process is important to ensure proficiency through the assessment; however, there is no gold standard for this [40, 41]. There are many approaches to standard setting, and one must determine whether the performance will be viewed in the context of minimal levels of achievement, such as with high-stakes examinations, or whether the purpose of the simulation is for the attainment of mastery. If the latter, the standard is set to relevance or importance rather than a normative score. Another approach is to provide formative rather than summative evaluation, which completely changes the concept of standard setting. In addition, a performance standard might be loosely defined in the case of an exploratory study. Nevertheless, frames of reference should be established, at least based on expert opinion. Once a performance reference can be established, data can be obtained, analyzed, and triangulated. For example, data from known groups (novices, experts, etc.) can be compared. Our research has revealed distinct differences between expert and novices’ performance, as evidenced by specific EEG markers [12] and kinematic biomechanical variables [14]. This allows automating the recognition of experts and novices through machine learning classification algorithms [12] or determining specific differences in psychomotor skills that can be incorporated into training design [14]. In addition, a reference can also be another commonly used metric, such as written assessment instruments that can correlate with objective data obtained through biological sensors or devices. Other measures that can be used as references include performance, anxiety, stress, or cognitive load indicators.

- Tip 8: Train the trainers and raters

Observational measures used in simulation-based medical education research, including high-stakes evaluations like licensing exams, are commonly prone to errors due to a lack of evaluator training and poor inter-rater reliability (IRR) [42]. We may assume that well-established tools with good reliability and validity evidence will yield quality data with high IRR; however, it is not always a guaranteed result [43]. The instructors and raters can often have varying levels of experience and diverse backgrounds, which may result in differences in their understanding of a simulation session’s objectives and targeted outcomes. Consequently, training the trainers and raters is essential. Feldman and Lazzara [42] suggest training recommendations for reducing rater errors and improving rater accuracy. Some of these recommendations include a planned rating training that considers the simulation scenario’s complexity and the raters’ clinical expertise. This should include a calibration process for raters to ensure the optimum level of validity and reliability, as well as the involvement of the raters during the planning process (many clinical teachers do not have simulation or educational backgrounds). It is essential to highlight that simulation educators often assume multiple roles. They may serve as raters while at the same time monitoring and running the overall simulation scenario, manipulating high-fidelity mannequins, and facilitating an effective feedback process [44]. Thus, trainers must have a comprehensive shared mental model surrounding all aspects of the simulation experience. One solution to help lower the trainer’s workload is to consider video-based assessments. The advantage of this is that the evaluation can be done asynchronously and by more than one person (allowing the establishment of IRR). However, the recording of all participants with high quality can be challenging.

## Focus on the Data and Its Potential for Impact

- Tip 9: Use appropriate methods to analyze the data

Multimodal data provides abundant, detailed information at an individual learner level, but it may be hard to conduct sample-based statistics. Although having an established hypothesis can facilitate the process, be open to some exploratory findings. Select a data management system that will help organize and store the data, considering ethical and privacy issues [45, 46] and ensuring easy accessibility and sharing among team members. Some research may call for synchronizing multiple data sources into one dataset. Diverse data streams present challenges that can hinder the analysis process, resulting in suboptimal results and conclusions. For example, the sensors of a simulation mannikin can provide important information regarding event logs that complement the observational instrument applied by the rater. However, without an adequate system and expertise, the benefits of integrating this information may not be seen, including the consolidation of actionable information for performance improvement. Automated data collection to a single management system avoids losing data; however, harmonizing the data streams into one big dataset is a challenging problem still ripe for development [47]. Having a data scientist on the team is important throughout this process. In addition, having team members with signal processing and perhaps machine learning and artificial intelligence expertise can help to make sense of the large volume of physiological data. Finally, consider the scope and limitations of the available data, including a limited number of participants due to the technical complexity, time, and other resources required to implement multimodal assessments in simulation.

- Tip 10: Leverage multimodal data for research and continuous improvement

One of the challenges with MMLA is the complexity of operationalizing multimodal data for research purposes [39]. It is essential to define how this data can contribute to research by fostering continuous improvement in simulation-based medical education. The aggregated data can help researchers test hypotheses, validate or revise established theoretical frameworks, explore new phenomena, and assess the overall impact of simulation-based training [48]. For example, multimodal data can inform researchers of common learning mistakes and optimize simulation logistics, such as tracking when mannequins need replacement or streamlining educational resources [49]. Additionally, MMLA data can support accreditation reporting systems by providing structured information to assess performance outcomes, thereby contributing to evidence-based simulation improvements [9]. Researchers can also utilize this data to refine the design of future simulation-based sessions by examining key performance indicators, such as task accuracy and timeliness. These insights create a foundation for understanding how learners perform in various scenarios, enabling targeted research into best practices for clinical skills development [50, 51].

### Teaching Note

While this tip focuses on research, the insights gained from MMLA can be invaluable for educators seeking to optimize student learning. Multimodal data can provide real-time, actionable feedback to learners, enhancing their ability to reflect on their performance and engage in deliberate practice [9]. Learning analytics dashboards (LADs), which visually organize key performance indicators, offer a powerful tool for presenting multimodal feedback in a format that supports personalized learning [48]. By delivering timely and trackable feedback, LADs help learners identify areas for improvement, shortening the learning curve for clinical skills acquisition [49]. Educators can leverage these research insights to facilitate continuous improvement in learner performance by integrating MMLA feedback into ongoing teaching strategies.

## Conclusions

Over the years, simulation-based medical education has proven to be an effective teaching modality. However, current assessment metrics rely on subjective sources, and teaching pathways are not always strategic. MMLA offers an opportunity to enhance the current simulation training practices. In recent years, data collection technologies and advanced machine learning approaches have developed exponentially. These advancements bring us to an exciting juncture to open the door for future disruptive ideas to take simulation-based learning and research to new heights. Multiple assessment modalities are more likely to elucidate gaps and challenges related to trainee competencies within dynamic clinical settings. Recent studies have effectively incorporated multimodal assessments into simulation-based education, utilizing various technologies to gather nuanced data. For example, sensors have been employed to track student location and movement within simulated clinical environments. Wearable devices have facilitated the collection of physiological information, such as heart rate and skin conductance, to assess student stress and engagement. Additionally, high-fidelity manikins have automatically recorded task completion, providing objective performance metrics. These existing applications highlight the feasibility and potential of multimodal assessments in enhancing simulation-based education. Consequently, multimodal assessment, aligned with the challenges of the learning environment, learner characteristics, and the learning gap, will yield valuable insights to evaluate educational efficacy and tailor instruction to optimize learning outcomes. Furthermore, this article promotes the integration of interdisciplinary teams and collaboration between researchers and clinical teachers with expertise in education, psychology, physiology, and simulation, and, on the other hand, data scientists, engineers, and learning and cognitive scientists. We propose practical considerations for successfully conducting multimodal assessments during simulation sessions. These recommendations are consolidated in Table 1 for a more practical application. Despite the described benefits, there is still much to explore in this vein, and this article is intended to be a resource for those interested in pursuing multimodal assessments during simulations.

**Table 1:** Checklist for developing a research project with multimodal assessment

**Tip**	**Steps**	✓
1. Establish a clear conceptual framework from the beginning	Define the educational gap/problem	
	Define the learner population	
	Define the educational context	
2. Conduct a thorough literature review	Systematically search, appraise, and synthesize existing research	
	Identify research gaps and methodological limitations	
	Identify literature-informed variables affecting this problem	
3. Assemble an interdisciplinary team	Determine required skills	
	Identify potential team members with interdisciplinary expertise	
4. Align research objectives and ensure ethical practice	Align research objectives with assessment framework	
	Obtain IRB approval or exemption	
	Ensure data management integrity (storage, anonymization, accuracy)	
5. Define outcome measures and data collection methods	Define educational objectives and outcomes	
	Establish performance indicators and hypotheses	
	Select multimodal data sources (e.g., video, surveys, observation)	
	Identify required signals and devices (e.g., EEG, eye-tracking)	
	Explore the use of automatized techniques	
	Pilot test and assess feasibility of the chosen methods	
6. Design simulation scenario	Align scenario with educational and research objectives	
	Ensure coherence and feasibility of educational objectives	
	Structure scenario events for efficient data collection and analysis	
	Create a timeline for scenario implementation and data collection	
	Maintain scenario realism and flow	
	Conduct pilot testing for scenario refinement and feasibility	
	Consider multimodal data calibration, synchronization, integration	
7. Establish a standard-setting process	Determine feasibility of standard-setting method	
	Select and define standard-setting method (if applicable)	
	Establish frames of reference (expert opinion, known groups, etc.)	
	Validate with objective data (EEG, eye-tracking, simulator sensors)	
8. Train the trainer and raters	Consider raters’ experience, similarities, and differences	
	Train the raters on scenario and assessment measures	
	Ensure inter-rater reliability (e.g., calibration, consensus)	
	Consider using video-based performance assessment	
9. Use appropriate methods to analyze the data	Define data management, storing, and synchronization protocols	
	Integrate multimodal data to address study hypotheses	
	Leverage technology for automated data collection	
	Select analysis methods tailored to research question and study design	
	Acknowledge and address data analysis limitations	
10. Leverage multimodal data for research and continuous improvement	Define how multimodal data will inform research and education	
	Use multimodal data to test hypotheses and explore new phenomena	
	Analyze data to optimize simulation logistics and resource allocation	
	Support accreditation reporting and evidence-based improvements	
	Explore harnessing multimodal data to personalize learning pathway	
